# The Simplification of Colour-Matching

**Published:** 1914-04-04

**Authors:** 


### X-RAY DOSAGE—A NEW RADIOMETER.

### The Simplification of Colour Matching.

The use of *x*-rays for therapeutic purposes in hospital practice is rapidly increasing. Judging from some of the results one sees in cases that have been epilated for ringworm, it is evident that the methods employed for the exact measurement of *x*-ray dosage are either unsatisfactory or difficult to use except by those of large experience. Sabouraud's pastilles are still largely employed and are wonderfully reliable in action, but the difficulty has been in the judging of the tints obtained, especially in the region of the colour scale where lies tint B or the epilation dose. Long practice is needed before a beginner can feel certain that he is matching the colours correctly. *X*-ray workers, therefore, will be glad to hear of any new method which will simplify this colour-matching, and at the same time provide accurate standards for comparison. The radiometer devised by Dr. Dudley Corbett, of St. Thomas's Hospital, will remove many of the difficulties that are commonly experienced in judging the tints given by the pastille, and provides reliable standards for the epilation dose and its fractions, which can be used in daylight or artificial light. The instrument has been in regular use at St. Thomas's Hospital for several months, and the results in ringworm have been most satisfactory.

### The Standards Employed.

In a recent paper to the *Lancet* Dr. Corbett calls attention to the fact that the tints attached to the Sabouraud-Noire card are not always the same, some representing a dose that will just epilate and no more, others an almost dangerous dose for unfiltered rays. The tint B, which has been standardised in this instrument, allows a margin of error of 20 per cent. on either side. In other words, 4/5 B on this scale will nearly always epilate, while 1 1/5 B is the limit of safety. The methods employed in the experimental work have been fully described elsewhere. The colour changes in the pastille itself when exposed to *x*-rays were worked out by means of a Lovibond tintometer, an apparatus for accurately measuring colours of every description. By combinations of tinted glasses of known spectroscopic value the tint of the pastille could be exactly matched from the unexposed state to a point when two B doses had been given. The results thus obtained were correlated in actual clinical practice on cases of ringworm, and the tint B finally standardised so as to allow the margin of error mentioned above.

The point of importance, therefore, about the standards employed in this radiometer is that they are composed of the Lovibond standard glasses in combination; their colour composition never varies, and they are easily kept clean. There are no paper standards to fade or get dirty. Further, there is a separate series of standards for working with daylight or with electric light, for the colour changes differ according to the character of the incident light. The standard electric light is that from an 8 c.p. carbon filament lamp with frosted glass; it is not advisable to work with any other means of illumination.

The apparatus itself consists of an oblong box divided by a central partition so that on looking down the eye-piece one can see the white background through small circular apertures. On the one side, level with the background, is a fitting to take the pastille in its holder, on the other is a groove in the instrument itself for the insertion of the standard glasses. The colour of the pastille as seen by reflected light can then be compared with that obtained by transmitted light through the standard glasses against the white background. It is possible to get such an exact match that one could not say on which side the pastille is situated. A difference of 1/5 B or 1 H is easily perceived.

As to whether it is easier to work by daylight or artificial light is a personal matter. In either case, it is obvious when using this instrument that the pastille fades very nearly as quickly under electric light as it does under daylight. One should practise with both methods. The following precautions should be observed. In daylight work in a good white light, avoid shadows and yellow light of any kind. In electric light use the standard lamp already mentioned, so arranged with a suitable shade that the pastille is situated eight inches from the lamp. The shade should be black, not coloured. No other light should be allowed to reach the pastille during examination. Any lamp should be discarded when the light becomes yellow with age. In both cases examine quickly to avoid fading the pastille. When it is desired to reach an accurate 1 B dose, put up the 4/5 B standard first; it is then easy to calculate how much more exposure is required for the extra 1/5.

### A Point of Importance.

It is important to see that none of the unexposed green colour of the pastille is visible through the small aperture; this will upset the reading. For very accurate work new pastilles should be used, as a bleached pastille never returns exactly to its old tint. When such a pastille is exposed to *x*-rays the colour changes will start a little further down the curve, and so the tint will require to be taken a little further than the normal tint for a given dose. It is a very slight increase, but is nevertheless appreciable and may amount to 5 to 10 per cent. But even here, if the margin of error is ample in the neighbourhood of the B tint, and if a pastille is not used more than three times and is well bleached in daylight after each exposure, serious errors are not likely to occur.

The standards usually provided are those in common use—namely, ¼, 1/3, ½, 4/5, 1/1, 1 1/5 and 2 B, but it should be noted that standards can be made up from any other dose that may be required.

In conclusion:—

(1) The experimental work has shown the colour changes occurring in the pastille when exposed to *x*-rays.

(2) By this means a permanent standard for tint B has been established which matches the pastille exactly, does not fade, is easily kept clean, and can be reproduced accurately in any quantity as it is standardised spectroscopically. It will therefore remain constant as long as the emulsion remains unaltered.

(3) Any fraction or multiple of this dose can be supplied up to 2 B.

(4) Either daylight or electric light can be used.

(5) The optical instrument itself, by cutting off extraneous light, greatly assists the colour comparisons, so that the error should never exceed 10 per cent.

(6) Dr. Corbett recommends that the epilation dose for ringworm should be taken as lying between 4/5 B and 1 B on his scale. Using the "fire position" method, 4/5 B is given over every area except that on the top of the head, where 1 B dose should be delivered. Using these accurately measured doses it is possible to get complete epilation with very little erythema and no dermatitis.

